# Association of Nighttime Speed Limits and Electric Scooter–Related Injuries

**DOI:** 10.1001/jamanetworkopen.2023.20868

**Published:** 2023-06-29

**Authors:** Rasmus Liukkonen, Heljä Aarnikko, Pekka Stenman, Sanna Ovaska, Aleksi Reito

**Affiliations:** 1 Center for Musculoskeletal Diseases, Tampere University Hospital, Tampere, Finland; 2 Tampere University, Faculty of Medicine and Health Technology, Tampere, Finland; 3 Unit of Traffic System Planning, City of Tampere, Tampere, Finland

## Abstract

This cohort study investigates whether nighttime speed restrictions are associated with the incidence of electric scooter–related injuries in Finland.

## Introduction

Electric scooters (e-scooters) are associated with a high risk of injuries, especially injuries occurring at nighttime or under the influence of substances.^1^ Several nighttime restrictions have been attempted to decrease the number of injuries, but the outcomes of those restrictions have been evaluated only without the use of user data.^2,3^ In addition, only limited data have been reported about the user database incidences of e-scooter–related injuries.^4,5^ Hence, we aimed to examine whether nighttime speed restrictions are associated with the incidence of e-scooter–related injuries.

## Methods

In this retrospective cohort study, we identified all the patients admitted to the emergency department of Tampere University Hospital (Tampere, Finland) between April 24, 2019, and September 30, 2022. The hospital is the only trauma center providing 24-hour emergency services for its catchment population of approximately 550 000 inhabitants. In addition, we requested user data (number of rides and kilometers driven) from the e-scooter companies. The Tampere University Hospital’s research director approved the study and waived the need for informed consent due to the study’s retrospective nature. We followed the STROBE reporting guideline.

In 2022, the nighttime speed restrictions were used in Tampere, as the maximum speed was electronically limited by the e-scooter companies to 15 km/hour between 12 am and 6 am. Hence, the summertime (June 1 to August 31) incidences were compared to evaluate the association of nighttime restrictions with incidence.

A search for e-scooter–related injuries was performed using related keywords to identify patients (eAppendix in Supplement 1). After identifying possible cases, electronic health records were examined manually for each of the patients. Injuries were categorized according to their location and severity. The severity was evaluated with the Abbreviated Injury Severity scale. Incidences were calculated with Poisson distribution and reported with 95% CIs. All the analyses were performed using the epitools package from R version 4.0.3 (R Project for Statistical Computing) from December 2022 to January 2023.

## Results

A total of 654 patients (mean [SD] age, 28.7 [10.6] years; 382 [58.4%] male) experienced an e-scooter–related injury during the summer periods between 2019 and 2022. During those periods, a total of 3 556 929 rides with 7 287 027 km of distance were driven. The mean total incidence was 18.39 (95% CI, 17.00 to 19.58) injuries per 100 000 rides and 8.97 (95% CI, 8.30 to 9.69) injuries per 100 000 km driven (Table 1).

**Table 1.** Characteristics of Electric Scooter–Related Injuries, Stratified by Year

Characteristic	Injuries, No. (%)			
	2019	2020	2021	2022
No. of injuries	76	111	222	245
Daytime injuries	36 (47.4)	55 (49.5)	117 (52.7)	135 (55.1)
Nighttime injuries	40 (52.6)	56 (50.5)	105 (47.3)	110 (44.9)
AIS severity ≥3 injuries	<5^a^	8 (7.2)	11 (5.0)	19 (7.8)
Age, mean (SD), y	30.5 (9.8)	28 (8.0)	27.9 (12.2)	28.9 (10.2)
Sex				
Male	45 (59.2)	71 (64.0)	124 (55.9)	142 (58.0)
Female	31 (40.8)	40 (36.0)	98 (44.1)	103 (42.0)
Operative treatment	<5^a^	10 (9.0)	14 (6.3)	27 (11.0)
Substance misuse				
No	34 (44.7)	30 (27.0)	89 (40.1)	107 (43.7)
Yes	33 (43.4)	60 (54.1)	73 (32.9)	69 (28.2)
Delayed presentation to ED	9 (11.8)	21 (18.9)	60 (27.0)	69 (28.2)

Abbreviations: AIS, Abbreviated Injury Scale; ED, emergency department.

^a^ Because of Finnish legislation, frequencies less than 5 cannot be reported as exact values.

The mean injury incidence was lowest in 2019 (16.94 [95% CI, 13.34-21.20] per 100 000 rides) and highest in 2020 (25.0 [95% CI, 20.56-30.10] per 100 000 rides). The injury incidence was similar in the years 2021 (17.75 [95% CI, 15.75-20.58] per 100 000 rides) and 2022 (17.34 [95% CI, 15.23-19.65] per 100 000 rides). The mean distance per ride decreased every year, and hence the mean injury incidence per 100 000 km increased almost 2-fold from 5.63 (95% CI, 4.43-7.04) in 2019 to 10.43 (95% CI, 9.16-11.82) injuries per 100 000 km in 2022 (Table 2).

**Table 2.** Summertime Incidence of Electric Scooter–Related Injuries^a^

Variable	2019	2020	2021	2022^b^
Total No. of injuries	76	111	222	245
Total No. of rides	448 734	444 402	1 250 719	1 413 074
Total distance driven, km	1 350 427	1 219 129	2 366 355	2 349 897
Mean distance per ride, km	3.01	2.75	1.89	1.66
Injury incidence (95% CI)				
Per 100 000 rides	16.94 (13.34-21.20)	25 (20.56-30.10)	17.75 (15.75-20.58)	17.34 (15.23-19.65)
Per 100 000 km driven	5.63 (4.43-7.04)	9.10 (7.49-10.96)	9.38 (8.31-10.86)	10.43 (9.16-11.82)

^a^ The summertime period was June 1 to August 31 during each of the study years.

^b^ Nighttime (12 am to 6 am) speed restriction was set to 15 km/h in the summer of 2022.

## Discussion

To our knowledge, no previous study has examined the association between nighttime restrictions and the incidence of e-scooter–related injuries. A limitation of this cohort study is its retrospective nature, which could bias our results. However, we evaluated the association of nighttime restrictions with the user database incidence of e-scooter–related injuries. On the basis of our results, the nighttime speed limit was not associated with reduced injury incidence. The results from our study can be used as reference values to evaluate the efficacy of new interventions. Although e-scooter–related nighttime injuries are a concern, it seems that the nightly speed limits might not be effective enough to substantially reduce them.

## Notes

Supplement 1. **eAppendix.** Search Terms

Supplement 2.
Data Sharing Statement
