# Supplementary material for: Association of Nighttime Speed Limits and Electric Scooter–Related Injuries
Source: JAMA Netw Open. 2023 Jun 29;6(6):e2320868. doi: 10.1001/jamanetworkopen.2023.20868 (PMC10311383; doi:10.1001/jamanetworkopen.2023.20868)
Supplement: Supplement 1. — eAppendix. Search Terms [file jamanetwopen-e2320868-s001.pdf]

Supplemental Online Content

Liukkonen R, Aarnikko H, Stenman P, Ovaska S, Reito A. Association of nighttime speed limits and electric scooter–related injuries. *JAMA Netw Open.* 2023;6(6):e2320868. doi:10.1001/jamanetworkopen.2023.20868

eAppendix. Search Terms

This supplemental material has been provided by the authors to give readers additional information about their work.

© 2023 Liukkonen R et al. *JAMA Network Open.*

eAppendix. Search Terms

Following search terms were used:

sähköpotku* *skuut* *e-skoot* *potkulau

Terms are in Finnish language. *Sähköpotkulauta* means electric scooter. Sometimes term *e-skootteri* is used as a direct translation from English word e-scooter. *Skuutti* is a vernacular word for e-scooter.

All EHRs from recent years are stored in an operational data store (ODS) format. An SQL query was run with the above search terms in the ODS database. The search was done word by word in all relevant specialty notes. These specialties were emergency medicine, orthopaedics, neurosurgery and intensive care.

Each matched record in the database was manually abstracted to see if it involved an e- scooter related injury.

© 2023 Liukkonen R et al. *JAMA Network Open.*
