# Supplementary material for: Association of Nighttime Speed Limits and Electric Scooter–Related Injuries
Source: JAMA Netw Open. 2023 Jun 29;6(6):e2320868. doi: 10.1001/jamanetworkopen.2023.20868 (PMC10311383; doi:10.1001/jamanetworkopen.2023.20868)
Supplement: Supplement 2. — Data Sharing Statement [file jamanetwopen-e2320868-s002.pdf]

Data Sharing Statement

Liukkonen. Association of Nighttime Speed Limits and Electric Scooter–Related Injuries. *JAMA Netw Open*. Published June 29, 2023. doi:10.1001/jamanetworkopen.2023.20868

Data
Data available: No

Additional Information
Explanation for why data not available: According to Finnish legislation, individual patient-level data cannot be made available to third parties.
